# Prevalence of exercise-induced bronchoconstriction in teenage football players in Tunisia

**DOI:** 10.4103/0256-4947.55318

**Published:** 2009

**Authors:** Imen Aissa, Amine Frikha, Habib Ghedira

**Affiliations:** a Department of Respiratory Diseases III, Abderrahman-Mami Hospital, Ariana, Tunisia; b Department of Institute of Sports and Physical Education, Manouba, Tunisia

## Abstract

**BACKGROUND AND OBJECTIVES:**

Studies on exercise-induced bronchoconstriction (EIB) in team sports are lacking. The aim of this study was to screen for EIB among amateur teenage football players in Tunisia and to compare EIB prevalence between regions.

**METHODS:**

One hundred ninety-six male football players (mean age [SD], 13.5 [0.5] yrs), practicing in three different cities of Tunisia (Tunis, Sousse and Sfax), underwent an outdoor free run of 7 minutes. Forced expiratory volume in one second (FEV_1_) was recorded prior to and at 0, 3, 5, 10, 15, 20 and 30 minutes after the run. Players were screened for EIB positivity defined as a greater than 10% decline in FEV_1_ from the resting value at any timepoint.

**RESULTS:**

FEV_1_ decreased more than 10% in 30% of the players. EIB positivity was more common in Sfax (15.8%) than in Tunis (7.7%) (*P* =.03). Air humidity during the study was higher in Tunis.

**CONCLUSION:**

EIB is prevalent among amateur teenage football players in Tunisia. The prevalence differs between regions and seems to be dependent on air humidity levels.

Exercise-induced bronchoconstriction (EIB) also referred to as exercise-induced airway hyperresponsiveness in athletes, is a common, but often unrecognized condition because it is underdiagnosed, occurring in both known asthmatic and healthy athletes.^1^ Sparsely studied, the EIB is defined as the ocurrence of a transient airway obstruction, immediately to 30 minutes after vigorous exercise, while exercise-induced asthma is used to describe this condition in subjects who have previously diagnosed respiratory symptoms.^1^,^2^ EIB affects 4% to 20% of the general population and 40% to 90% of asthmatic patients.^1^,^3^ The prevalence of EIB among athletes trained for high level endurance competitions (cross-country skiers, ice skaters, cyclists, swimmers or long distance runners) ranges from 10% to 55%.^1^–^4^ EIB in amateur athletes has not been well documented, but was reported at a lower prevalence of 5.3% in amateur endurance-trained athletes.^5^

The pathogenesis of EIB is not clearly defined, but there is general agreement that inhalation of large volumes of cold, dry air during exercise leads to a loss of heat and water from the bronchial mucosa leading to airway cooling and drying.

The variability in prevalence of EIB depends on the type of exercise, environmental conditions, diagnosis criteria and a previous history of asthma.^8^ In Tunisia, asthma prevalence in the teenage non-athlete population has been estimated at 13.2% in Tunis city,^9^ but EIB prevalence in teenage athletes has not been evaluated.

The aim of the present study was to estimate the prevalence of EIB and assess the effect of climate on prevalence in teenage high level amateur football players in different regions of Tunisia using a submaximal free running test.

## PATIENTS AND METHODS

Paticipants included 196 young male amateur football players in three different regions of Tunisia: 99 in Tunis in the north, 59 in Sousse in the center part of the country and 38 in Sfax in the south. Their mean (SD) age was 13.45 (0.51) years, ranging from 13 to 14 years old. The median range was 13.45. All had 3 to 4 years of training experience. The subjects and their coaches gave verbal consent to study participation. The study was carried out in the afternoon (3 to 6 PM), the coldest part of the day, from December 2002 to March 2003. Age, height and weight were recorded for each footballer.

Pulmonary function testing and measurements were carried out at the training and competition sites of the different teams and under usual environmental conditions. Prior to a test session, all players completed a 10-item medical history questionnaire that focused on asthma symptoms or medication taken, atopy, and EIB symptoms (Appendix 1). Then, each footballer had to perform a submaximal free outdoor running test of 7 minutes. Continuous monitoring of heart rates was obtained with a handheld pulse-meter. Pulmonary function tests were assessed according to the American Thoracic Society (ATS) guidelines10 with a flow-volume spirometer (heated pneumotachograph, Medikro 909, Medikro Oy, Finland) and FEV_1_ was determined at each tested time point. Baseline spirometry was followed by an exercise challenge performed in groups of five. Each subject was asked to perform a 2 minute run by increasing the running speed until the heart rate reached 80% to 90% of the predicted maximum heart rate, calculated as 200 minus the age in years, and then asked to maintain this speed over 5 minutes. FEV_1_ was recorded at rest and at 0, 3, 5, 10, 15, 20 and 30 minutes after the exercise, and the lowest value from three trials was chosen to represent the FEV_1_ in the participant. Predicted values were assessed from spirometric reference values in Tunisian children.^9^ EIB was defined as a fall in FEV_1_ of at least 10% from the resting value at any time point after exercise, based on ATS guidelines.^10^ The percent variability of FEV_1_ at each time point was computed as follows:

[(rest FEV_1_–lowest post-exercise FEV_1_)/rest FEV_1_]×100.^10^

Measurements of temperature and air humidity were obtained from the national weather center, in the three different cities.

Descriptive statistics are presented as mean and standard deviation. The chi-squared test with Yates correction and non-parametric test of Wilcoxon was used for percentage comparisons, and the *t* test for means comparisons. The level of significance was selected as *P*≤.05.

## RESULTS

All subjects completed the study protocol. Relative humidity was higher in Tunis comparing with Sfax and Sousse (83% vs 65% and 68%) (Table 1). There were no reported signs or symptoms of underlying chronic asthma or EIB. No medication use was reported by the participants. The prevalence of EIB was 30% (59/196) based on a 10% fall in FEV_1_. There was no significant difference between football players with and without EIB with regard to age and weight, but subjects with EIB were significantly shorter than negative EIB counterparts (Table 2). Resting FEV_1_ expressed in liters per minute was compared to reference values in both groups according to the subject's height established by the following equation: ln (FEV_1_)=constant+height coefficient×(height)^10^ The frequency of mean FEV_1_ at baseline was not different between subjects with and without EIB (*P*=.2) (Table 2).

**Table 1:** Weather conditions during the study.

	Mean temperature (°C)		Mean humidity (%) Mean temperature (°C)	
	December	January	December	January
**Tunis**	14.2	12.6	83	81
**Sousse**	15	13.4	68	68
**Sfax**	14.2	12.6	65	65

**Table 2:** Anthropometric and baseline lung function data of subjects with exercise-induced bronchoconstriction (EIB) and controls.

	ElB	No ElB	*P* value
Patients n (%)	20 (10.2)	176 (89.8)	
Age (yr)	13.45 (0.51)	13.43 (0.49)	.86
Weight (kg)	50.65 (9.1)	54.00 (8.9)	.68
Height (cm)	161 (0.07)	165 (0.07)	.016
Baseline mean FEV_1_ (%)	91.6 (5.34)	95 (4.4)	.2

Data are presented as mean (SD) unless otherwise indicated. FEV_1_: forced expiratory volume in one second.

**Figure 1:**
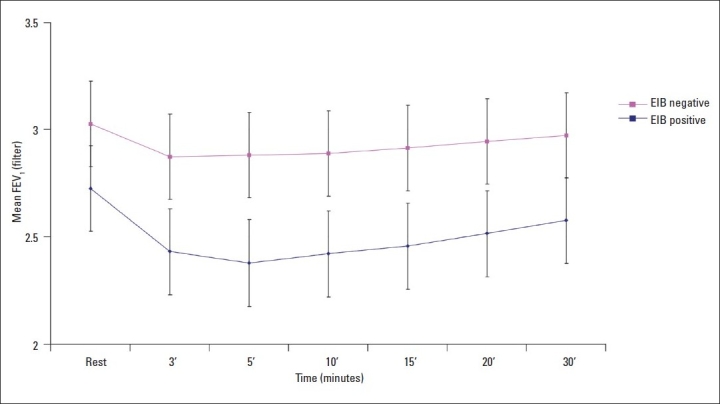
Distribution of FEV_1_ before and at different times after exercise (0, 3, 5, 10, 15, 20 and 30 min) for football players with or without exercise-induced bronchoconstriction (EIB).

A fall in FEV_1_ was observed in all subjects; 49% had a fall in FEV_1_ between 5% and 10% of the rest FEV_1_ value. Intergroup comparisons of FEV_1_ values showed that subjects with EIB had lower FEV_1_ values at rest and at all time points following exercise when compared to EIB negative subjects (Figure 1). EIB-positive subjects experienced significant declines in FEV_1_ after exercise. The fall was maximal between the third and fifteenth minute but remained significantly lower than EIB-negative subjects at 30 minutes post exercise.

**Figure 2:**
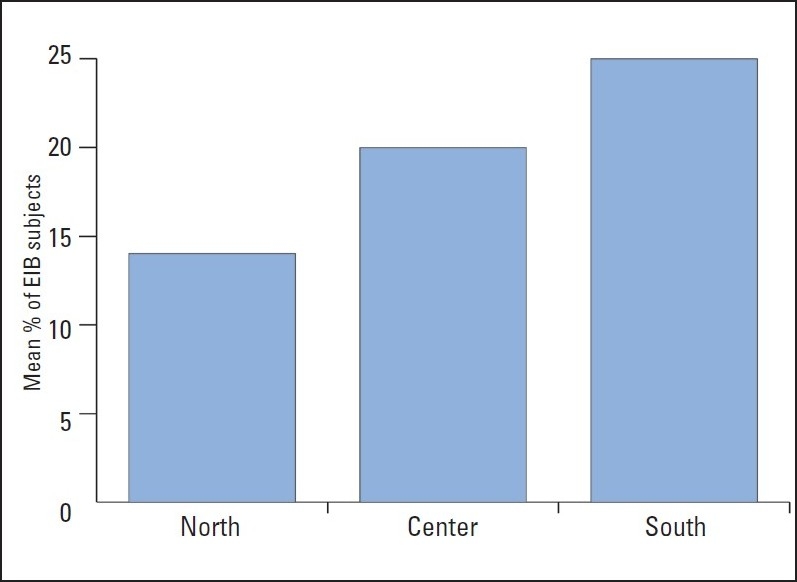
EIB prevalence in different regions of Tunisia.

The prevalence of EIB differed in the three regions, with the lowest rate in the Tunisian team (n=14, 23%) and the highest in the Sfaxian team (n=25, 42%) (*P*=.03) (Figure 2).

The time points at which the greatest decline of FEV_1_ occurred in patients with EIB ranged from 3 to 15 minutes, and the distribution was as follows: 20% experienced the greatest decline at 3 minutes postexercise; 40% at 5 minutes; 25% at 10 minutes; and 15% at 15 minutes postexercise.

## DISCUSSION

The current study found a high prevalence (30%) of EIB in apparent healthy football players in Tunisia. The prevalence varied between regions in Tunisia depending on the climate. The high prevalence of EIB is not surprising since numerous studies on highly trained athletes have reported a relatively high prevalence of EIB (Table 3). Initial reports on EIB among athletes included the surprising finding of an 11% prevalence among the US 1984 summer Olympic team athletes.^11^ In football players, the prevalence of EIB ranged from 9% to 50%.^12^–^17^ Kukafka et al^14^ found a 9% prevalence of EIB among non-asthmatic young football players. A prevalence of up to 50% was reported in subjects who frequently complain of exercise-induced respiratory symptoms.^15^

**Table 3:** Prevalence of EIB/EIA among athletes from published studies.

Authors (Ref)	Method	Condition	Population: Prevalence
Voy^12^	Questionnaire	EIA, asthma	American Olympic summer athletes: 11%
Mannix^18^	Exercise test	EIB	Competitive figure skaters: 35%
Feinstein^16^	Exercise test	EIB	Male football players: 19%
Sodal^17^	Methacholine BHR	BHR	Female soccer players: 35.5%
Ross^23^	Reversibility test	Reversibility	Canadian football players: 56%
Schoene^13^	Exercise test	EIB	Male athletes: 10%
			Female athletes: 26%
Kukafka^14^	Exercise test	EIB	Young football players: 9%
Brudno^22^	Exercise test	EIB	Scholars: 47%

EIA: exercise-induced asthma, EIB: exercise-induced bronchoconstriction, BHR: bronchial hyperresponsiveness.

The prevalence of EIB may be influenced by the value of the decline in FEV_1_ used to define EIB.^18^ Authors agree that to diagnose EIB, a decrease in respiratory flow of at least 10% from baseline is recommended.^12^,^17^–^20^ To increase specificity, others have employed a more stringent criterion of at least a 15% fall.^14^,^21^,^22^ In 1999, the ATS put guidelines for methacholine- and exercise-challenge testing criteria^10^ recommending a fall in FEV_1_ of more than 10%. Bronchodilator reversibility to an inhaled β_2_-agonist should be documented after a lung function measurement.^2^,^23^ This test was not used in this study, since no player had clinical symptoms and the purpose of this study was not to diagnose exercise-induced asthma, but to assess the prevalence of EIB.

In this study, the comparisons of baseline FEV_1_ were made according to spirometric reference values established for healthy Tunisian children.^24^ Subjects in the present study with EIB were significantly shorter than EIB negative counterparts; in fact, height as a surrogate for smaller lungs and therefore smaller airways may be an important correlative factor to explain EIB prevalence in shorter subjects, in the opinion of the present authors. Concerning the lack of asthma signs reported in this study, we propose the hypothesis that young footballers selected to compete in national teams might not report symptoms of asthma or EIB since asthma suspicion often leads to exclusion by the coach.

Different challenge tests can be used to assess EIB. Pharmacological challenge tests, such as methacholine challenge, have low sensitivity for EIB diagnosis.^25^–^27^ Sport-specific exercise in the field with respect to the standardization of both the workload and the environmental conditions of temperature and humidity is recommended in athletes.^27^ Nevertheless, the gold standard to assess EIB in elite athletes is the eucapnic voluntary hyperpnea (EVH) with dry air.^26^ An alternative to the EVH challenge is the hyperosmotic challenge test, either with hypertonic saline or inhaled mannitol dry powder, which have both a high sensitivity (96%) and specificity (92%) for EIB.^25^,^26^

EIB prevalence may vary with seasons.^1^ It is well documented that EIB is more prevalent when using an exercise challenge with cold dry air and during pollen season.^3^,^15^,^28^–^30^ For this reason, we conducted this study during winter and part of spring.

Relative humidity is an important factor contributing to EIB occurrence.^31^ The difference in the EIB among subjects in different cities in different parts of the country can probably be explained by humidity being consistently lowest in the southern region (by nearly 20%) vs the northern region. Tunisia is located on the Mediterranean Sea, and has high rates of humidity throughout the year, so humidity levels recorded were higher than those recommended by the American Thoracic Society (50%), which in itself may protect against EIB, so the reported 30% rate of EIB may be an underestimation.

In conclusion, our findings suggest that EIB may be common in athletes. The prevalence difference observed between regions may be explained by different levels of humidity. Screening for EIB and therapeutic follow-up are reasonable considerations for athletes to train at the necessary intensity and to be able to achieve peak performance.

## Appendix 1: Medical history questionnaire.

1. Do you smoke cigarettes, cigar, or pipe?
2. Does anyone in your family have asthma?
3. Does anyone living in your household smoke at home?
4. Are you allergic to inhalants allergens, such as dust, pollen, or animal hair?
5. Do you take any medications on a regular basis (steroids, birth control pills, etc)?
6. Have you ever been told that you have asthma or exercise-induced asthma, even as a child?
7. Have you ever had wheezing, chest tightness, itchy eyes or nose?
8. Do you have trouble with breathing or would you experience coughing after running 1 mile and resting for 10 or 15 min?
9. Have you ever missed school, work, or practice because of chest tightness or cough or wheezing or prolonged shortness of breath?
10. Do you have trouble breathing or do you cough during or after activity?
